# Societies and Transactions

**Published:** 1897-07-31

**Authors:** 


### SOCIETIES AND TRANSACTIONS.

The great value of the communications to some of our medical societies, as they appear in the Transactions, is in striking contrast with the extremely select and even attenuated character of the audiences before which some of them have been read. The *Practitioner* gently twits the Royal Medical and Chirurgical Society on the scanty attendances at its meetings, and suggests that, if affairs go on at the present rate much longer, they will come to be like the exercises for the medical degree at Oxford in the early part of the Queen's reign, when the candidate read the prescribed lectures to the Bedell! Well, we have not got quite to that pass yet, and it must be remembered that this great society offers other attractions to its Fellows besides its meetings. Yet one cannot but feel that these reunions have about them a dash of dulness which is quite unnecessary. It is, in fact, difficult to understand why a society which is sufficiently in touch with modern methods to print its papers beforehand, so as to make a reasonable discussion possible, and which even goes so far as to distribute an abstract at the meeting, should be so tied down by tradition as to insist on having the whole of each communication read out in all its weary details to those present. It is the attempt to kill two birds with one stone, and to make the same paper do both for the meeting and the Transactions, which is at the root of the difficulty. Not for worlds would we do ought to diminish the value of the Transactions; but when the whole paper has been printed, and is already in the hands of the Fellows, we cannot but see that the interest of the meetings would be vastly increased if the author would be good enough in a short ten minutes, and not more, to state just the point he wants to raise. We quite recognise that any society which trusts to spoken addresses tends to become superficial, but so long as the papers themselves are printed and are offered for criticism that objection falls to the ground. If the papers were taken as read, and the subject were shortly introduced by the author, practically the whole meeting would be occupied by discussion—which, after all, is the only really interesting part. Leading men will go to discuss, but they will not go to be lectured at. And such lectures! The very fact that they are written for the Transactions disqualifies them for public delivery.

